# Development of an Internet-Based Cognitive Behavioral Therapy Self-Help Program for Arabic-Speaking Immigrants: Mixed-Methods Study

**DOI:** 10.2196/11872

**Published:** 2018-12-18

**Authors:** Tomas Nygren, Matilda Berg, Ali Sarkohi, Gerhard Andersson

**Affiliations:** 1 Department of Behavioural Sciences and Learning Linköping University Linköping Sweden; 2 Department of Clinical Neuroscience Karolinska Institutet Stockholm Sweden

**Keywords:** internet, cognitive behavioral therapy, Arabs, focus groups

## Abstract

**Background:**

Recent years have seen an increase in Arabic-speaking immigrants in Sweden and other European countries, with research showing this group to suffer from elevated levels of various forms of psychological disorders. There is a lack of treatment options for immigrants with mild to moderate mental health problems, with barriers including lack of accessible services and concerns that problems will not be understood by health care providers.

**Objective:**

This study aims to describe the process of developing a transdiagnostic internet-based cognitive behavioral therapy self-help program in Arabic for mild to moderate symptoms of common psychological problems such as anxiety, depression, and insomnia.

**Methods:**

The iterative development process, including feedback from 105 pilot users as well as 2 focus groups, is described.

**Results:**

Overall, the modules were rated as acceptable by the pilot users, with overall ratings ranging from 3 to 4 points on average for the respective modules on a 5-point Likert scale. Feedback from the 2 focus groups was overall positive with regard to the content and structure of the program but also included suggestions for improving the Arabic translation as well as the usability of the material.

**Conclusions:**

An internet-based self-help program that is deemed acceptable by an Arabic-speaking audience can be successfully developed, thus providing increased access to psychological help for an at-risk population. However, further research regarding the efficacy of this type of intervention is warranted.

## Introduction

### Background

Recent years have seen a sharp increase in Arabic-speaking immigrants in Sweden, with a majority of these arriving from either Syria or Iraq [1]. Research has found that this group suffers from elevated levels of various forms of psychological disorders [2-4], most likely because of both pre- and postmigration factors, such as exposure to armed conflict and trauma in their country of origin, as well as the stress and uncertainty inherent in the asylum-seeking process [5]. Although research on the mental health of Arabic-speaking immigrants in Sweden specifically is scarce, existing studies indicate an increased risk for depression, anxiety, and psychosis compared with the majority population [2].

There is a lack of treatment options for immigrants with mild to moderate mental health problems in Sweden, partly because of an overall low availability of psychotherapy in Swedish primary care [6], and especially given the fact that many recently arrived immigrants naturally lack proficiency in the Swedish language, restricting access even further [7]. Moreover, previous research has established that immigrant populations tend to underutilize mental health services in relation to the majority population [8], with common barriers including lack of services in their respective language (eg, Arabic), concerns that problems will not be understood by health care professionals because of cultural differences, as well as stigma associated with mental health issues [9]. This was also true in a Swedish study, which found that refugees received more psychiatric inpatient care than the Swedish native population but similar levels of outpatient care, leading the authors to speculate that there were barriers to accessing care for milder forms of psychiatric problems [10].

Internet-delivered cognitive behavioral therapy (ICBT) could be one way to approach some of the issues mentioned above, as internet-delivered treatment can be translated into different languages and has the potential to reach more people than conventionally delivered psychotherapy, requiring less therapist resources [11]. Moreover, it has been hypothesized that internet-delivered treatment could increase treatment seeking for groups with higher mental health stigma by offering increased anonymity and accessibility [12].

Importantly, any treatment is more likely to be effective if it is *culturally adapted* to fit the target audience [13], with cultural adaptation referring to the process of adapting or substituting one or more aspects of a treatment to make it more aligned with the target population’s norms and values [14]. Chu and Leino [14] outlined a data-driven framework for the adaptation of psychological interventions, providing common concepts and a taxonomic structure for adapted treatment components. They distinguished between adaptations of peripheral treatment components, referring to engagement and treatment delivery, and core treatment components, referring to adaptations of the actual treatment components themselves [14].

At least two randomized controlled trials have tested culturally adapted ICBT, one targeting depression among Chinese Australians [15] and the other targeting posttraumatic stress disorder among Arabs living in the Middle East [16]. Both trials showed moderate-to-large between-group effects on primary outcome measures. However, both studies tested forms of *therapist-guided* ICBT, that is, ICBT where the patient has access to a therapist that provides weekly support and also answers questions about the treatment material, treatment techniques, as well as the application of those techniques. Therapist-guided ICBT has been shown to be more effective than *self-guided* ICBT, which refers to treatments where there is no therapist contact [17]. However, a recent meta-analysis by Karyotaki et al [18], analyzing data from 3876 participants in trials of self-guided ICBT for depression, revealed a significant between-group effect compared with the control conditions with a between-group effect size of Hedge *g*=0.27, a small but significant effect, leading the authors to conclude that self-guided ICBT can be considered an evidence-based first-step approach in treating symptoms of depression [18].

### Objectives

On the basis of these previous findings, an ICBT self-help program in Arabic aimed at Arabic-speaking refugees and immigrants living in Sweden with mild to moderate mental health problems was developed. This study reports on the process of developing this program.

## Methods

### Point of Departure

**Figure 1:**
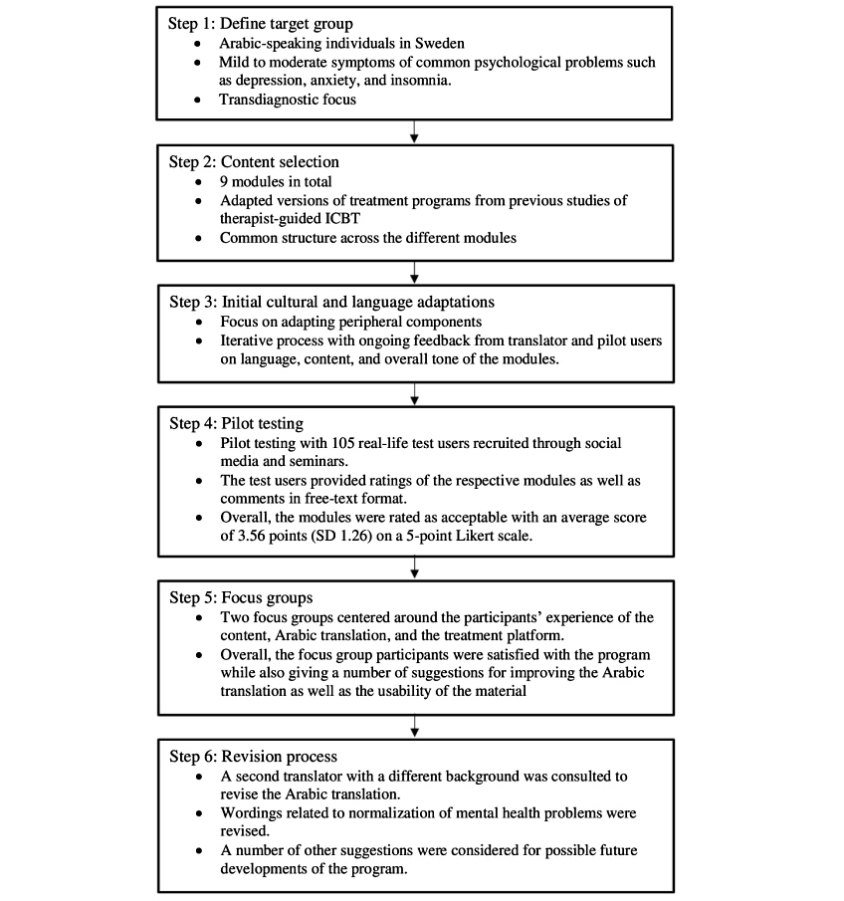
Flowchart of the steps involved in the developmental process. ICBT: internet-delivered cognitive behavioral therapy.

The development of the self-help program was initiated in collaboration with the Swedish Association of Local Authorities and Regions. The core development team involved 4 clinical and research psychologists, a webmaster, and a translator. The Iterapi platform [19] was used to deliver the program. The program was made available in 3 languages: Swedish, English, and Arabic. The focus here will be on the development of the Arabic version of the program. For an overview of the developmental process, see Figure 1.

### Target Group, Aims, and General Considerations

The target group was Arabic-speaking individuals living in Sweden with mild to moderate symptoms of common psychological problems such as anxiety, depression, stress, and insomnia. The aim was to develop a first-step approach to treating these symptoms in a cost-effective way that would allow for wide dissemination. Hence, a decision was made to develop a self-guided rather than therapist-guided program. In addition, we made the program transdiagnostic, with modules targeting a variety of different expressions of psychological problems, rather than just focusing on a specific disorder. This decision was motivated by previous research indicating that transdiagnostic ICBT programs are equally effective in treating anxiety as disorder-specific programs and possibly more effective in treating depression as well as improving quality of life [20].

### Content Selection

It was decided that the program should consist of 9 modules in total: (1) Introduction, (2) Do you feel depressed? (3) Do you have problems with anxiety? (4) Do you have trouble sleeping? (5) Do you feel stressed out? (6) Do you have problems with worrying and rumination? (7) Do you have difficulty managing your emotions? (8) Do you suffer from painful memories? and (9) Final chapter and summary. The first chapter included a general introduction to basic cognitive behavioral therapy (CBT) principles and the last chapter a general summary and strategies for maintaining and continuing positive changes. The contents of the remaining 7 chapters were largely adapted versions of treatment programs from previous studies by our research group (see eg, [21,22]) that were abbreviated and adapted to fit the self-help format, for example, by removing some of the more interactive elements that were deemed to require ongoing therapist support. The basic structure of the modules was similar across the different problem areas, starting with psychoeducation about the problem area, a CBT conceptualization exemplified with a case example, and finally a number of exercises or strategies for handling maladaptive thoughts and behaviors viewed as causing and maintaining the problems.

### Cultural and Language Adaptation

The culture and language adaptation consisted of an iterative process, with ongoing feedback from the translator as well as number of pilot users of mixed Arabic origin commenting on the language, content, as well as the overall tone of the modules. The next step involved a total of 105 real-life test users evaluating 133 modules in Arabic through a built-in evaluation function at the end of each module. The users were asked to rate the respective modules on a 5-point Likert scale with regard to whether they found the content understandable, whether it was helpful, and whether they intend to make use of the information and advice in the future. They were also asked to provide an overall rating of the module using the same 5-point Likert scale. It was also possible to provide comments in a free-text format. In addition, 2 focus groups were held, with the aim to provide detailed feedback with regard to how the modules are experienced by the target audience.

The focus groups centered around three main questions:

- How did the participants experience the content and structure of the modules?
- How did the participants experience the Arabic translation?
- How did the participants experience the treatment platform?

The focus group data were analyzed using qualitative content analysis [23], focusing on identifying key themes in the participants’ experience of the treatment program.

Importantly, with regard to cultural adaptation, the initial versions of the program focused mainly on the adaptation of peripheral treatment components including language, semantics, as well as the treatment format itself, which was hypothesized to enhance treatment seeking and engagement by increasing anonymity, given that mental health is still highly stigmatized in Arabic culture [24]. With regard to adaptation of core treatment components, the initial versions of the modules contained no adaptations of this kind other than trying to normalize psychological problems in the context of having recently gone through stressful changes and upheavals (such as fleeing one’s country of origin) as well as modifying case examples to make them more appropriate for the target audience. One reason for this is the fact that Arabic culture is very diverse across the Arab world [25], making it difficult to adapt the treatment in a way that would be suitable for Arabs originating from different regions of the Arab world. In addition, Hassan et al [26] reported a changing discourse regarding mental health among Syrian refugees more compatible with a Western viewpoint, illustrating the fact that culture is a dynamic phenomenon [27] and thus making it impossible to a priori know to what extent Western psychological treatment models need to be adapted for Arabic immigrants in Sweden, hence the need for a more iterative adaptation process. Along this line, a study by Beshai et al [28] failed to find any differences between depressed Canadians and Egyptians with regard to negative and positive automatic thoughts and dysfunctional attitudes, lending support to the applicability of the cognitive model of depression in an Arab population.

### Participants

#### Test Users

The test users evaluating the Arabic versions of the modules were recruited through advertising through social media platforms and Web search engines. In addition, a number of Web-based seminars aimed at professionals working with recently arrived immigrants were held, aiming to disseminate information about the project. Interested individuals visited the program website where they had to register using an email address and provide a personal password. Once registered, the participants had free access to all the material and were asked to provide ratings as described above at the end of each module. No information was collected about the participants, except for their ratings of the respective modules. The reason for not collecting additional information about the test users was to guarantee their anonymity, as we hypothesized that this would make it significantly easier to recruit test users, given the aforementioned mental health stigma in Arabic culture.

#### Focus Groups

The participants in the first focus group consisted of, to our knowledge, the only Arabic-speaking clinical psychologist and the only 2 Arabic-speaking psychology students living in the county of Östergötland where the project took place. All 3 participants were female and were aged between 20 and 40 years. All 3 participants were born in Arabic-speaking countries and had immigrated to Sweden as children together with their families. The participants in the second focus group were recruited at the local branch of the Swedish Red Cross language group for recently arrived immigrants. The 6 participants comprised 5 recently arrived Arabic-speaking immigrants from 4 different countries (Sudan, Eritrea, Afghanistan, and Somalia) with varying professional backgrounds as well as 1 librarian with experience of working with Arab immigrants volunteering for the Swedish Red Cross. All 5 immigrants were male, whereas the librarian was female, with the participants aged between 20 and 60 years.

## Results

### User Evaluations

The results from the 105 real-life test users are summarized in Table 1. Overall, these users rated the modules as acceptable. However, because of the small number of users evaluating some of the modules, it is difficult to evaluate the relative acceptability of the individual modules. With regard to comments by test users in the free-text format, only 21 of the users made use of this function. Of these, 5 users commented on the helpfulness of the program, stating that it was easy to understand or that they preferred this treatment format compared with visiting the doctor. One of these users also asked about the possibility to translate the material into additional languages. Furthermore, 5 other users were more ambiguous or negative in their comments, with 1 user questioning the usefulness of this type of text-based approach. Two others expressed difficulties in applying the material, whereas 1 of the 2 simultaneously expressed gratefulness for an increased theoretical understanding gained from reading the texts. Another user explicitly stated that he or she wanted a quick solution and that the program failed to provide this. The remaining users used the free-text comment section either to ask a specific question regarding their psychological or medical problems or to make more general statements (eg, about mental health), and thus, they did not provide specific feedback on the material per se.

### Focus Groups

Overall, three main themes emerged from the analysis of the first focus group, which were as follows: *Word choices in the Arabic translation*, *Computer literacy in target population*, and *Satisfaction with content and structure.* With regard to the first theme, all participants agreed that some parts of the Arabic translation needed revision, as some words were overly academic and therefore might be difficult to understand for nonacademics. They also agreed on other words being primarily used in North African countries and therefore making some paragraphs confusing to Arabs originating from countries in the Middle East. In addition, the participants also pointed out a number of spelling and grammatical errors in need of correction.

**Table 1:** Mean rating and SD for treatment modules in Arabic.

Module	Did you find content understandable?, mean (SD)	Did you find the material helpful?, mean (SD)	To what degree will you make use of this information?, mean (SD)	Overall rating, mean (SD)	Recommend to a friend, n (%)
Introduction (n=52)	3.67 (1.42)	3.44 (1.42)	3.58 (1.46)	3.5 (1.2)	45 (87)
Do you feel depressed? (n=38)	4 (1.41)	3.61 (1.5)	3.3 (1.56)	3.55 (1.35)	32 (84)
Do you have problems with anxiety? (n=4)	3.5 (1.73)	2.5 (1.73)	2.5 (1.91)	3.25 (1.71)	2 (50)
Do you have trouble sleeping? (n=9)	3.89 (0.92)	4.11 (1.05)	4.33 (0.71)	3.44 (1.51)	7 (78)
Do you feel stressed out? (n=4)	4.5 (0.58)	4.5 (0.58)	4.5 (1)	4 (1.41)	4 (100)
Do you have problems with worrying and rumination? (n=14)	4 (1.3)	3.71 (1.44)	3.64 (1.45)	3.93 (1.27)	12 (86)
Do you have difficulty managing your emotions? (n=5)	3.8 (1.01)	2.8 (1.3)	3.6 (0.89)	3.2 (1.48)	4 (80)
Do you suffer from painful memories? (n=4)	4.75 (0.5)	3.75 (0.5)	3.75 (0.5)	4 (0)	3 (75)
Final chapter and summary (n=2)	4 (1)	2.5 (0.5)	4 (1)	3 (0)	2 (100)
Overall (n=132)	3.88 (1.33)	3.54 (1.4)	3.57 (1.43)	3.56 (1.26)	111 (84.1)

The second theme pertained to whether or not Arabs living in Sweden had sufficient computer literacy skills to make use of the program. This was primarily brought up as a concern by 1 participant who gave the suggestion to have a short video clip on the start page, introducing the user to the Web page and material to make the program more user-friendly. With regard to the third theme, all participants agreed that they were satisfied with the content and structure of the material and saw little need for improvements or revisions.

The analysis of the second focus group resulted in four main themes, which were as follows: *Overall satisfaction with program*, *Enhancing usability*, *Stigmatization of mental health*, and *Additional content*. With regard to the first theme, all participants in the second focus group agreed that the overall structure and content were satisfactory, although they said that the program could be enhanced by making the material more interactive, for example, by adding free-text fields, which users could use to reflect on their own progress with the exercises. The second theme had to do with several suggestions put forward by the participants on how to improve the usability of the program. Similar to the first focus group, they too pointed out several spelling and grammar errors in need of correction to make the texts easier to understand. One participant also recommended having shorter versions of the material for users with less reading experience, whereas another participant suggested that it would be good to have the possibility to have the text read out loud by the program for users with reading or concentration difficulties. The third theme focused on stigmatization of mental health problems in Arabic culture and ways to address and work around this to get more users to actually make use of the program. Relating to this, 1 participant suggested putting more emphasis on biological explanatory models for mental illness as this might be viewed as less stigmatizing than psychological explanations. Another participant suggested putting even more emphasis on normalizing psychological problems and help seeking to reduce stigmatization. A third suggestion from another participant was to have a person with authority in an Arab population, such as a psychiatrist or psychologist with an Arabic background, introduce the material in a short video clip at the opening page, dispelling common myths and misunderstandings about mental health. Overall, there was a concern among the participants that mental health stigmatization would constitute a barrier for many users accessing the program. Finally, the fourth theme had to do with some participants seeing a need for additional content in the program to meet the needs of Arab immigrants in Sweden. One participant suggested we address problems with concentration, perhaps in a separate module. An additional suggestion concerned having more information with regard to good habits when living in Sweden, such as eating vitamin D supplements and the importance of going outside despite the cold weather. Finally, 1 participant also saw a need among many immigrants to address issues relating to shame and guilt concerning culture-specific practices (eg, culture-specific sexual practices and child-rearing practices) that come about when viewing these practices from the lens of Swedish culture.

### Revision Process

In response to the feedback received, it was decided that a second translator would be consulted to further review the Arabic translation as this was a theme that was brought up in both focus groups. This translator was herself an immigrant from Syria with an academic background from Damascus, whereas the first translator was born in Sweden and had acquired Arabic as a second language and was currently living in Egypt. With regard to suggestions to increase usability by shortening the material even more, this was decided against, given the potential risk to dilute the content, especially given that the material already consisted of distilled versions of well-researched programs. However, other suggestions relating to this theme, such as having a function that reads the text out loud, were deemed interesting but outside the scope of the current revision process. With regard to the issue of stigmatization as a barrier to accessing the material that was brought forward by the second focus group, we decided to look over the wordings of the program in relation to normalization of mental health problems. However, in most cases, we decided to stick with the original wording as we did not want to blur the important distinction between clinically significant suffering and normal functioning. Moreover, we also decided not to change the emphasis more to biological explanatory models as we felt that this might risk making the CBT conceptualization less conceptually clear and stringent. Regarding some of the other themes that were brought up in the focus groups, such as adding new content, addressing other problem areas, or using video to introduce the material to make it more user-friendly and possibly reduce stigmatization, we felt that these were interesting ideas for future developments of the program, given their more encompassing nature.

### The Final Internet-Based Self-Help Program

The final version of the program was equally accessible on computers and mobile devices such as mobile phones and tablets. To make use of the material, the user had to register using an email address and provide a personal password. No personal information was collected about the user except their ratings of the various modules. The program is currently freely available through the Iterapi platform at Linköping University, as described above, and the plan as of now is to continue to host the program through this platform.

## Discussion

### Summary of Findings

Overall, it seems that an acceptable internet-based self-help program in Arabic can be successfully developed, based on the results from the focus groups and test user evaluations. With regard to the cultural adaptation of the program, it seems that the relatively modest adaptations made to the material were sufficient to make the program acceptable for the target audience. This might reflect a changing discourse regarding mental health, both in the Middle East and among Arab immigrants, moving from more traditional, religious, or supernatural explanatory models to biological and psychological models more in line with a contemporary Western perspective [26].

### Discussion of Results

The 105 test users gave acceptable ratings for the modules, although the sample size was too small to examine the relative acceptability of the different modules. With regard to the comments left by the users in the free-text section, they can be seen as indicative of the fact that this type of intervention will likely be helpful for some but not all individuals, as reflected by the small effect sizes found in previous research [18]. Other individuals will likely need more extensive treatment approaches where more help is provided, for example, with regard to the application of treatment principles.

With regard to the 2 focus groups, although they both agreed on the overall acceptability of this program, they also had a number of suggestions for improving the program. In the end, we chose to mainly focus on one of these suggestions, namely, consulting a second translator to review the wording of Arabic translation, removing or changing words that were overly academic or that are only used in North African countries. However, the participants in the second focus group also came with a number of interesting suggestions on how to further develop the program, which we have considered below under Future Directions.

### Limitations

In retrospect, it would have been a good idea to conduct focus groups earlier in the developmental process before deciding on which problem areas to focus on in the different modules. Had we done this, we might have included additional material aimed at concentration difficulties, as suggested in the second focus group. In line with this, it might also have been beneficial to conduct the focus groups and revise the program before testing the program with a group of test users. The reason that we chose the procedure that we did was to first get a sense of the overall acceptability of the program and then, if the program overall was deemed acceptable, get more specific feedback through the focus groups. However, reversing the order would have enabled us to have the test users try out the final version of the program, which would likely have yielded different feedback and also somewhat higher ratings of acceptability.

An additional limitation concerns the lack of information regarding sociodemographic characteristics of the test users. This limits the generalizability of the findings, given that we cannot know for sure whether the test users are representative of the target population. On the other hand, as there was no monetary compensation or other compensation for the test users, it is likely that the individuals who signed up for the program did so because they had some degree of mental health problems that they wanted help with. Furthermore, given that the program will continue to be freely accessible in the same way as during the test period, it is reasonable to assume that the test users are representative of those who might use the program in the future.

A final limitation concerns the fact that online programs such as this one necessarily are limited to those individuals with access to the internet, which is probably not always the case for newly arrived immigrants or refugees. However, it is important to underscore that internet-based self-help programs merely constitute 1 potential pathway for accessing psychological help and should not be used as an excuse to not develop other types of services or treatment formats.

### Future Directions

Future directions include working to disseminate the program further, making it widely known to both service providers and potential users so that it is actually put to use. Other possible future directions include going forward with some of the suggestions put forward by the 2 focus groups, such as having voice recordings of the material, integrating the use of video more in the material, and addressing problems with concentration, as mentioned above. Another interesting possibility would be to test the material in a more formalized way in a randomized controlled trial, to evaluate the efficacy of the program in treating mental health problems among the target population.

### Conclusions

In conclusion, an acceptable internet-based self-help program in Arabic for mild to moderate symptoms of psychological problems can be successfully developed. However, further research is warranted regarding the efficacy of the program. Future developments of the program should consider making the program more user-friendly by, for example, adding voice recordings of the material as well as adding additional content addressing specific concerns of the target population.

## Abbreviations

CBT: cognitive behavioral therapy
ICBT: internet-delivered cognitive behavioral therapy
